# Children and Young Adults Factor Merit Into Their Judgments of Gender‐Based Science Resource Inequalities

**DOI:** 10.1111/desc.70096

**Published:** 2025-11-16

**Authors:** Marley B. Forbes, Riley N. Sims, Melanie Killen

**Affiliations:** ^1^ Department of Human Development and Quantitative Methodology University of Maryland College Park Maryland USA

**Keywords:** gender, merit, moral judgments, resource inequalities, science

## Abstract

Inequalities in access to important resources and opportunities between social groups persist throughout societies worldwide. Social psychological research has shown that adults often use meritocratic beliefs to justify the existence of such inequalities. Yet, the developmental origins of meritocratic beliefs have yet to be fully explored. This study investigated how children and young adults (*N* = 144; 5‐ to 6‐year‐olds, *M* = 5.83, *SD* = 0.97; 9‐ to 11‐year‐olds, *M* = 10.74, *SD* = 0.68; 18‐ to 22‐year‐olds, *M* = 19.92, *SD* = 1.10) factored information about merit into their moral judgments and reasoning about science education resource inequalities between groups of girls and boys. Confirming our hypotheses, participants overall judged inequalities that disadvantaged high‐merit groups more negatively than inequalities that disadvantaged low‐merit groups, regardless of which gender group was disadvantaged. Further, exploratory analyses revealed age‐related differences in judgments of inequalities that disadvantaged girls, but not boys. Whereas all age groups judged inequalities that disadvantaged boys more negatively when boys were described as high‐merit compared to low‐merit, only older children judged inequalities that disadvantaged girls more negatively when girls were described as high‐merit compared to low‐merit. Age‐related differences also emerged for participants’ reasoning about inequalities, such that older children were more likely to reason about merit, and less likely to reason about equality, compared to both younger children and young adults. These novel findings offer insights into how concerns for merit shape individuals’ moral judgments of social inequalities throughout childhood and young adulthood.

**Summary:**

- Overall, children and young adults judged inequalities of science resources that disadvantaged high‐merit groups more negatively than inequalities that disadvantaged low‐merit groups.
- More positive judgments of inequalities were associated with a lower likelihood of reasoning about equality and a higher likelihood of reasoning about merit.
- Older children were more likely to reason about merit and less likely to reason about equality compared to both younger children and young adults.
- Exploratory analyses showed age‐related differences in the extent to which participants factored merit into their judgments of inequalities that disadvantaged girls, but not boys.

## 1 Children and Young Adults Factor Merit Into their Judgments of Gender‐Based Science Resource Inequalities

Social inequalities, or patterns of unequal access to important resources and opportunities between members of different social groups, persist throughout the global world. These inequalities exist across various domains of life (e.g., education, healthcare, workforce opportunities) and result in disparate outcomes for individuals from different gender, racial/ethnic, and socioeconomic backgrounds (Center for American Progress 2022; Economic Policy Institute 2017; Fiscella and Sanders 2016). The persistence of these inequalities contrasts with the wealth of evidence demonstrating humans’ early emerging orientations towards fairness, equality, and concerns for others’ welfare (Forbes et al. 2024; Vaish and Tomasello 2023). How is it, then, that many individuals tolerate, and at times even support, these harmful forms of inequality?

One well‐studied explanation for this apparent incongruity relates to meritocratic beliefs, or ideologies that frame individuals’ efforts and abilities as the main determinants of outcomes in life (Ledgerwood et al. 2011; Mijs 2021). Meritocratic beliefs can serve to justify existing social inequalities because they purport that one's position within a system of inequality is determined by factors within their control, and thus those who occupy disadvantaged positions do so because of their own shortcomings (Jost et al. 2015). This justification has been referred to as the “myth of meritocracy” because attributing inequalities solely to differences in individuals’ efforts and abilities neglects the ways in which historical and systemic patterns of exclusion and discrimination contribute to existing inequalities (Bonilla‐Silva 2022). Furthermore, meritocratic beliefs can facilitate biased and discriminatory attitudes towards disadvantaged social groups, which can in turn be used to legitimate social inequalities (Madeira et al. 2019; McCoy and Major 2007).

While this framework has typically been applied to adults’ justifications of broader social issues, such as wealth inequality, the origins of these meritocratic beliefs likely emerge far earlier in development. Research on children's conceptions of fairness in resource allocation contexts shows that, by 3 years of age, children accept and prefer unequal resource allocations that advantage and reward hardworking individuals over their non‐hardworking counterparts (Baumard et al. 2012; Schmidt et al. 2016; Noh et al. 2019). Notably, though, most studies that have revealed this early emerging preference for merit lack additional contextual factors that characterize social inequalities. For example, few studies have examined how children incorporate concerns for merit into judgments of inequalities of necessary resources (i.e., resources with implications for well‐being) and instead have mainly focused on luxury resources (i.e., resources that are nice to have but do not have implications for well‐being) (see Rizzo et al. 2016 for an exception). Additionally, no known studies have presented children with merit‐based inequalities of necessary resources between individuals or groups that vary in a real‐world social group membership (e.g., gender, race, wealth status). This intergroup component is a central aspect of social inequalities because stereotypic beliefs about certain groups can facilitate individuals’ merit‐based justifications of social inequalities.

A context in which concerns for merit and stereotypic beliefs may interact to shape views of inequalities across development is in science, technology, engineering, and mathematics (STEM). Women remain underrepresented in several STEM careers globally, which represents a form of social inequality because these careers are associated with benefits such as high wages and prestige, and women continue to have unequal access to these lucrative positions (Cheryan et al. 2025). While the causes of women's underrepresentation in STEM fields are complex, a robust body of research has highlighted how stereotypes, bias, and discrimination contribute to gender inequalities in these fields (Cheryan et al. 2017; Moss‐Racusin et al. 2018; Simon et al. 2017). Notably, these group‐related processes begin to influence inequalities long before individuals enter the workforce, with stereotypic beliefs contributing to the exclusion and unfair treatment of girls in STEM contexts beginning in childhood (McGuire et al. 2022, Mulvey et al. 2023).

What has yet to be examined is how concerns for merit may interact with group‐related concerns, namely stereotypic beliefs and associations, to uniquely shape judgments of gender‐based STEM inequalities. Given that many adult STEM fields are also characterized by meritocratic schemas (Blair‐Loy and Cech 2022; Garr‐Schultz et al. 2023), investigating how children coordinate these various factors in their judgments and reasoning about gender‐based STEM inequalities may provide novel insights into the developmental origins of meritocratic beliefs as a social‐cognitive framework that contributes to the persistence of gender STEM inequalities.

The aims of the present study were threefold. First, we sought to examine whether individuals in early childhood, late childhood, and young adulthood incorporated concerns for merit into their judgments of science resource inequalities. Specifically, did they view giving fewer resources to groups who showed high merit in science as less acceptable than giving fewer resources to groups who showed low merit? Second, due to the emergence of gender STEM‐related stereotypes in early childhood (Bian et al. 2017; Master et al. 2021), we investigated whether the application of concerns for merit to judgments of inequalities was consistent when different gender groups occupied different positions within inequalities. That is, did individuals view inequalities that disadvantaged high‐merit groups more negatively than inequalities that disadvantaged low‐merit groups when both boys and girls were disadvantaged by inequalities? Finally, we examined how individuals’ judgments of inequalities related to their prioritization of moral concerns for merit and equality in their justifications for their judgments.

### 1.1 Theoretical Framework: Social Reasoning Developmental Model

This study was motivated by the social reasoning developmental (SRD) model, which provides a framework for understanding how individuals coordinate moral and group‐related concerns when making judgments and decisions about what is fair in intergroup contexts (Elenbaas et al. 2020; Rutland and Killen 2017). Research guided by the SRD model has shown that negative judgments of intergroup inequalities often relate to prioritizing moral concerns (e.g., equality, others’ welfare), whereas positive judgments of intergroup inequalities often relate to prioritizing group‐related concerns (e.g., stereotypes, in‐group bias) (Cooley et al. 2019; Elenbaas et al. 2016; Sims et al. 2023).

However, in inequality contexts where information about recipients’ merit (i.e., effort, work contribution) is salient, inequalities that advantage high‐merit individuals or groups are typically preferred and judged as fair (Noh et al. 2019; Rizzo et al. 2020). This becomes more complex, though, when resources that have implications for others’ welfare are allocated meritoriously. A study by Rizzo et al. (2016) found that 3‐ to 8‐year‐olds evaluated merit‐based allocations of necessary (i.e., health‐related) resources more negatively than merit‐based allocations of luxury (i.e., play‐related) resources. Participants also referenced concerns for others’ welfare more in their reasoning for judgments of necessary resource allocations than luxury resource allocations (Rizzo et al. 2016). Thus, despite children's preferences for rewarding merit in resource allocation contexts (Baumard et al. 2012; Schmidt et al. 2016), they demonstrate advanced capacities for coordinating their concerns for merit with competing moral concerns for others’ well‐being.

Given that health‐related resources represent just one type of necessary resource, it is important to examine how concerns for merit shape children's inequality judgments involving other types of necessary resources, such as educational supplies. Previous research has established that children endorse equal rights to education and negatively evaluate the denial of access to education by late childhood, in part due to their recognition of how a lack of education negatively impacts children's well‐being (Helwig and Jasiobedzka 2001; Peterson‐Badali et al. 2004). However, the intersection of these beliefs and children's preferences for merit has yet to be examined in the context of resource inequality judgments. An aim of this study was therefore to examine whether children prioritized concerns for merit over equality and others’ welfare when judging the acceptability of inequalities of educational resources.

Education, like many major societal institutions, is rife with inequalities based on social group membership (Economic Policy Institute 2017), and thus, an educational resource inequality context presents the opportunity to also investigate how information about group membership is factored into individuals’ moral judgments. One specific form of inequality that is salient in education throughout development and has been found to affect later workforce inequalities is gender inequality in STEM contexts (Cheryan et al. 2025; Mulvey et al. 2023).

### 1.2 Merit and Gender Group Membership in STEM Contexts

STEM contexts are well‐suited for investigating how children and young adults factor concerns for both merit and group membership into their moral judgments because many STEM domains are simultaneously characterized by meritocratic schemas (Blair‐Loy and Cech 2022; Garr‐Schultz et al. 2023) and gender stereotypes and biases (Cheryan et al. 2017; Moss‐Racusin, Moss‐Racusin et al. 2018; Mulvey et al. 2023). By 6 years of age, children may endorse gender stereotypes about interests and abilities in STEM fields (Master et al. 2021; McGuire et al. 2022) and about brilliance and intelligence more broadly (Bian et al. 2017). Importantly, these stereotypes can influence children's peer interactions and contribute to the exclusion of girls in STEM contexts (McGuire et al. 2022; Mulvey et al. 2023). These early experiences of gender‐based exclusion serve as precursors to the sort of bias and discrimination that girls and women may continue to face in STEM throughout adolescence and adulthood (Rogers et al. 2021; Simon et al. 2017).

Due to the emphasis on merit, ability, and innate characteristics like brilliance in STEM fields (Blair‐Loy and Cech 2022; Meyer et al. 2015), it is unsurprising that meritocratic beliefs have been applied to justify existing gender STEM inequalities. For example, some individuals believe that women remain underrepresented in STEM domains because girls and women just aren't as interested in or good at STEM compared to boys and men (Charlesworth and Banaji 2019). This justification represents the “myth of meritocracy”, as it fails to acknowledge that women's underrepresentation in STEM fields is also due to the historical exclusion and discrimination they have faced in these domains. Furthermore, it exemplifies the ways in which meritocratic beliefs can interact with stereotypic thinking to justify inequalities. Recent research on individuals’ moral judgments of gender‐based inequalities of science resources has shown this pattern, with children and young adults using stereotypes and other group‐related concerns to support their positive judgments of inequalities that gave girls fewer science resources than boys (Sims et al. 2023).

Since research has not yet investigated individuals’ moral judgments of gender‐based STEM inequalities in contexts where information about recipients’ merit is also provided, the extent to which stereotypic associations may shape individuals’ beliefs about different gender groups’ inherent deservingness or claim over STEM resources remains unclear. Thus, another aim of this study was to investigate whether individuals’ prioritization of concerns for merit in their moral judgments of science resource inequalities was consistent regardless of the gender group disadvantaged by the inequality. In other words, does merit matter equally when both boys and girls are disadvantaged by inequalities, or do the strong cultural stereotypes associating boys with STEM more than girls interact with beliefs about merit to uniquely shape moral judgments?

### 1.3 Age‐Related Differences in Moral Judgments and Reasoning

Although the sensitivity to information about merit in resource allocation contexts and gender STEM stereotypes both begin to develop by early childhood (Baumard et al. 2012; Master et al. 2021; McGuire et al. 2022; Rizzo et al. 2016), there are several reasons to investigate how individuals from different age groups apply these concerns to their moral judgments and reasoning. From a theoretical standpoint, research on moral judgments in intergroup contexts has primarily focused on children and adolescents (Forbes et al. 2024), therefore leaving the developmental trajectory of decision‐making about resource allocation in intergroup contexts incomplete.

This is important because the SRD model proposes that individuals become increasingly adept at coordinating multiple factors into their reasoning and decision‐making as they get older (Elenbaas et al. 2020). Recent studies have found that as children enter adolescence and young adulthood, they are more capable of applying group‐related concerns, specifically knowledge about group status, to their moral judgments and decision‐making, such that they view inequalities that disadvantage historically disadvantaged groups as especially wrong (Herry et al. 2023; Killen et al. 2024; Yüksel et al. 2021). However, none of these studies included information about recipients’ merit. Given how important concerns for merit appear to be in inequality contexts from early childhood to adulthood, it is unclear whether age‐related differences in the extent to which individuals prioritize merit in their moral judgments and reasoning about necessary resource inequalities will arise.

There is also a rationale for investigating these questions with individuals from different age groups from an applied perspective, as understanding these developmental trajectories is critical for the construction of intervention efforts aimed at increasing gender equity in STEM. Specifically, different methods may be effective during distinct developmental periods depending on individuals’ social‐cognitive abilities. Finally, investigating these beliefs beyond childhood is important because the stakes of decisions involving the factors of merit and group membership become increasingly high later in life (e.g., academic admissions, hiring decisions, tenure promotions). Thus, this study included samples of younger children, older children, and young adults.

### 1.4 The Present Study

The present study investigated children's and young adults’ coordination of concerns for merit and group membership in their judgments and reasoning about a gender‐based resource inequality in a STEM context. Specifically, we asked younger children (5‐ to 6‐year‐olds), older children (9‐ to 11‐year‐olds), and young adults (18‐ to 22‐year‐olds) to judge the acceptability of science education resource inequalities between groups of boys and girls and to provide their reasoning for their judgments. Participants were presented with groups of boys and girls who (1) were either *advantaged* (had more resources) or *disadvantaged* (had fewer resources) regarding science education resources, and (2) were *high‐merit* (worked hard and had an interest in science) or *low‐merit* (did not work hard and had little interest in science).

We manipulated merit by varying groups’ amount of effort and interest in science due to previous research highlighting that, by the end of early childhood, children increasingly focus on effort and the positive intentions of acts when making judgments and decisions about how to fairly allocate resources (Noh et al. 2019). Thus, although effort and interest are distinct constructs, we paired them together because interest represents another positive intention that children may consider in relation to a group's deservingness of resources, especially in an educational context. Additionally, merit was manipulated at the group level to explore how concerns for merit and stereotypic beliefs related to gender group membership may interact to shape individuals’ judgments of the fairness of science education resource inequalities. The group‐level inequalities, therefore, mirrored the structure of many existing social inequalities, where access to important resources and opportunities differs for members of different social groups. Based on the SRD model (Elenbaas et al. 2020) and previous research on gender inequality in STEM contexts (Herry et al. 2023; Sims et al. 2023), we made the following predictions.

#### 1.4.1 Judgments of Inequality

We expected that female participants would judge inequalities more negatively overall than would male participants (H1). This hypothesis was motivated by prior research showing that children and adolescents from historically disadvantaged backgrounds tend to judge unfair treatment based on their disadvantaged identity (i.e., gender) more negatively than those from historically advantaged backgrounds (Cooley et al. 2019; Mulvey et al. 2024). We also expected that participants overall would judge inequalities more negatively when the disadvantaged group was high‐merit compared to low‐merit (H2) due to the importance of merit in individuals’ inequality judgments from early childhood to adulthood (Baumard et al. 2012; Ledgerwood et al. 2011; Mijs 2021; Schmidt et al. 2016).

We also expected age‐related differences in judgments of inequalities that disadvantaged girls. Specifically, we hypothesized that older children and young adults would judge inequalities that disadvantaged girls more negatively than would younger children (H3), and that young adults would judge inequalities that disadvantaged girls more negatively than would older children (H4). These hypotheses were motivated by previous research demonstrating that young adults judged science education resource inequalities that disadvantage girls more negatively than younger children (Sims et al. 2023), and by the SRD model's broader proposition that, with age, individuals increasingly apply their knowledge of a group's status to their moral judgments of specific inequalities that disadvantage that group (Elenbaas et al. 2020).

#### 1.4.2 Reasoning for Judgments of Inequality

Based on previous findings on the relations between children's resource inequality judgments and reasoning in contexts where merit was relevant (Rizzo et al. 2016; Rizzo et al. 2020), we expected that the more positively participants judged inequalities, the less likely they would be to use equality reasoning to justify their judgments (H5a). We also expected that participants would be more likely to use equality reasoning to justify their judgments of inequality when the low‐merit group was disadvantaged compared to advantaged (H5b).

Additionally, we expected that the more positively participants judged inequalities, the more likely they would be to use merit reasoning to justify their judgments (H6a). We also hypothesized that participants would be more likely to use merit reasoning to justify their judgments of inequality when the high‐merit group was disadvantaged compared to advantaged (H6b). Given mixed evidence on how children's reasoning about merit and equality varies by age in different contexts (Elenbaas et al. 2016, Rizzo et al. 2016; Schmidt et al. 2016), we did not have confirmatory hypotheses about age‐related differences for reasoning. However, age was still included in the reasoning analyses.

## 2 Method

### 2.1 Participants

Participants (*N* = 144) included three age groups: younger children (*n* = 49; 24 girls; *M*_age_ = 5.83 years, *SD*_age_ = 0.97 years), older children (*n* = 47: 24 girls; *M*_age_ = 10.74 years, *SD*_age_ = 0.68 years), and young adults (*n* = 48; 24 girls; *M*_age_ = 19.92 years, *SD*_age_ = 1.10 years). Participants were 52% White/European American, 15% Black/African American, 11% Multiracial/Multiethnic or other racial/ethnic group, 10% Asian/Asian American, and 7% Latine, with 5% declining to report. The sample included participants with average reported parental income between $120,000 and $150,000 across racial/ethnic groups. Children were recruited from elementary schools and after school programs in the Mid‐Atlantic region of the United States. Signed parental consent forms and child verbal assent were obtained for all participating children. Young adults were recruited from the SONA undergraduate participant pool from a public research university in the Mid‐Atlantic region of the United States. Participating young adults provided written consent and verbal assent. Data for this study were collected between April 2018 and June 2019. This study was not pre‐registered.

### 2.2 Procedure

**FIGURE 1:**
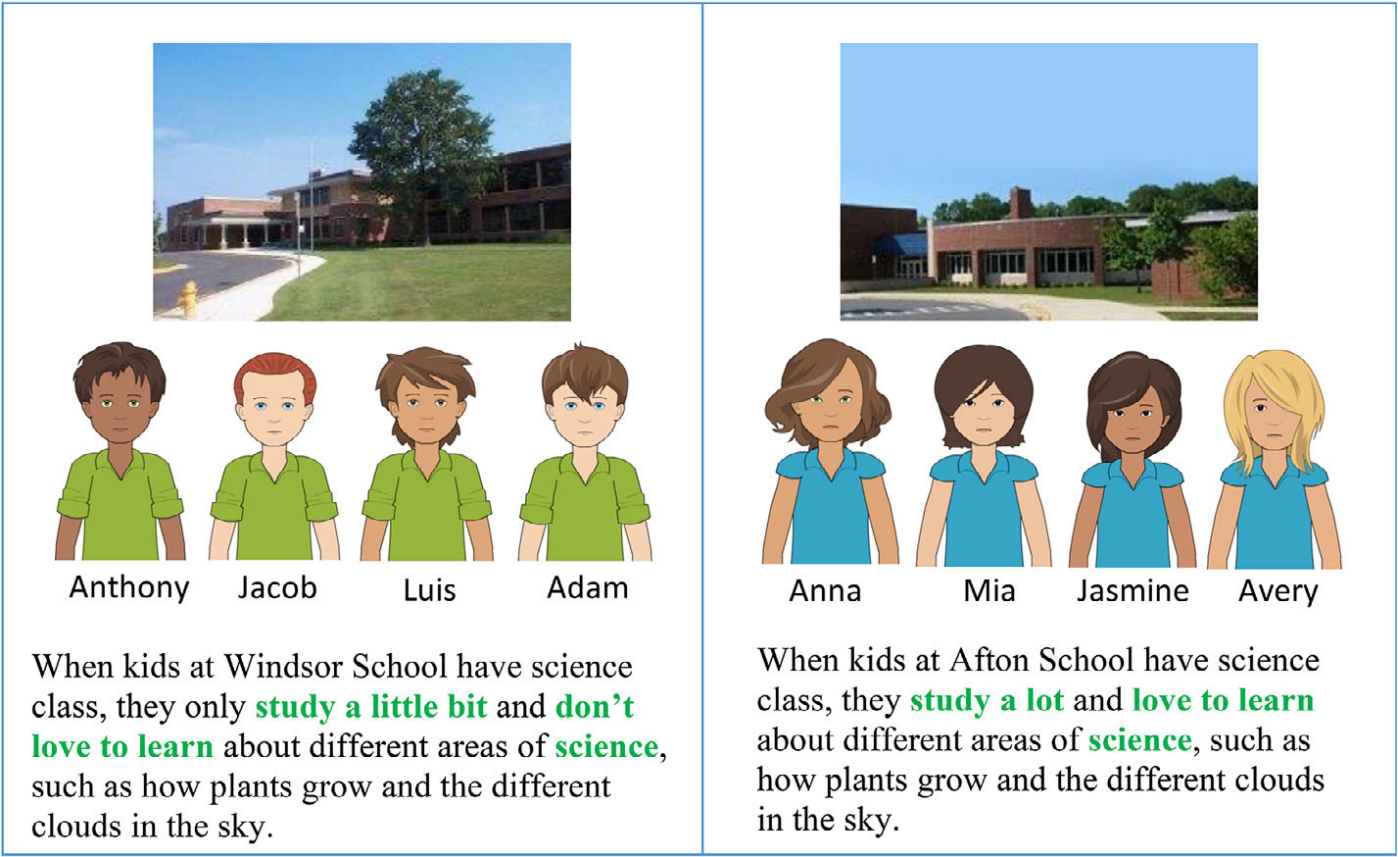
Presentation of the high‐ and low‐merit groups. This figure displays an example of the protocol where boys were the low‐merit group and girls were the high‐merit group.

Younger children were individually interviewed by a trained experimenter in a quiet space at their site. Each interview took approximately 25 minutes to complete. Older children and young adults independently completed the same protocol given to younger participants via a written survey, and trained experimenters were nearby to answer any questions. Participants were first introduced to two different schools in the same city. One school was shown to have only girls, and the other was shown to have only boys. Participants were then told about the merit level of each school. Students at high‐merit schools were described as those who “study a lot and love to learn about different areas of science”, and students at low‐merit schools were described as those who, “study a little bit and don't love to learn about different areas of science” (Figure 1).

**FIGURE 2:**
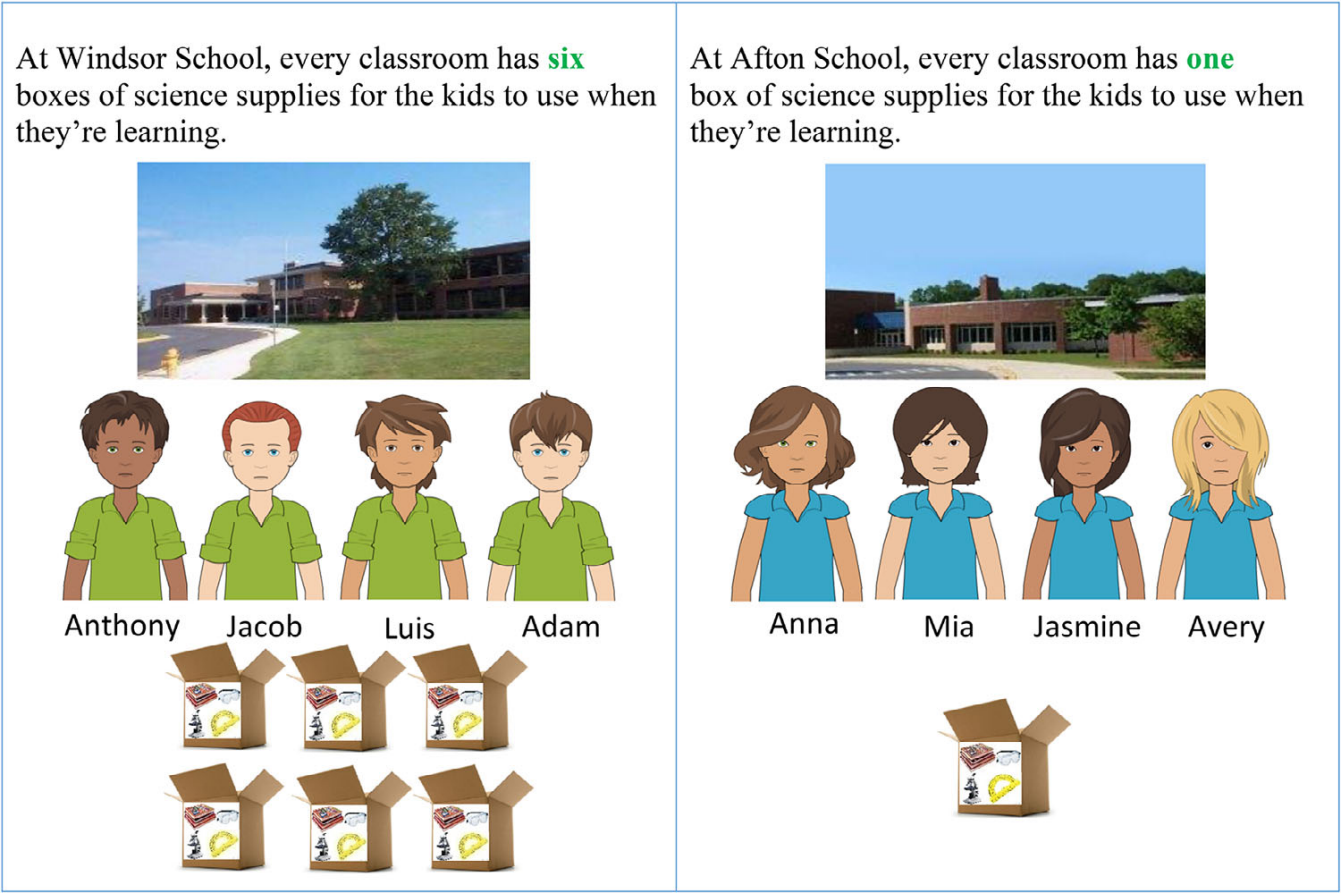
Presentation of the resource inequality. This figure displays an example of the protocol where boys were the advantaged group and girls were the disadvantaged group.

Next, a resource inequality was presented between schools such that students at one school had six boxes of science supplies to use when learning (i.e., advantaged group) and students at the other school had one box of science supplies to use when learning (i.e., disadvantaged group) (Figure 2). The science supplies were described to participants as “really important” several times throughout the study. Participants were reminded about the merit level of students at each school and were then asked to provide a judgment of the resource inequality between the schools. Finally, they were asked to provide reasoning for their judgment.

All participants viewed two vignettes. The gender of the students at the disadvantaged school was varied between‐subjects such that participants either viewed girls *or* boys as the disadvantaged gender group for both vignettes. Presentation of the groups as high‐ or low‐merit was varied within‐subjects such that all participants saw one vignette where boys were the high‐merit group and girls were the low‐merit group, and one vignette where girls were the high‐ merit group and boys were the low‐merit group. The names of the schools presented in the two within‐subjects conditions were different to make it clear to participants that these were new schools and not the same ones they viewed in the previous condition. Additionally, presentation of the two within‐subjects conditions was counterbalanced, and filler items were administered between the conditions to limit participant fatigue.

### 2.3 Measures

#### 2.3.1 Judgments of Inequality

To assess the acceptability of the resource inequality between the two schools, participants were asked, “How okay or not okay is it that [member of advantaged group's] school has more boxes of science supplies than [member of disadvantaged group's] school?” Participants responded on a 6‐point Likert‐type scale (1 = *really not okay*, 6 = *really okay*).

#### 2.3.2 Reasoning

Participants were asked to provide reasoning for their judgments. Based on previous research (Elenbaas et al. 2016; Rizzo et al. 2016), participant reasoning was coded into the following categories: (1) Equality (e.g., “All children should have the same access to education, including supplies”), (2) Merit (e.g., “Because the girls study a lot and they only have one box that is not fair”), and (3) Missing/Uncodable (e.g., “I don't know”). Final analyses were conducted utilizing equality and merit reasoning. Two independent coders who were blind to the hypotheses of the study coded 25% of the data (*n* = 36) and reached an inter‐rater reliability of Cohen's κ = 0.85.

### 2.4 Data Analytic Plan

Given the repeated‐measures nature of our design (i.e., each participant provided a judgment and reasoning about an inequality when boys were the high‐merit group *and* when girls were the high‐merit group), we used mixed‐effects models to analyze participants’ responses.

A linear mixed‐effects regression model was run to examine participants’ judgments of inequality. The model included fixed‐effects of gender, a gender group disadvantaged by inequality (girls, boys) X high‐merit group (girls, boys) interaction, an age group X gender group disadvantaged by inequality interaction, and a random intercept for participant.

Logistic mixed‐effects regression models were run to examine participants’ use of different reasoning categories to justify their judgments of inequalities. Separate models were run for each reasoning category (equality, merit). Both models included fixed effects of age group, judgments of inequality, a gender group disadvantaged by inequality (girls, boys) X high‐merit group (girls, boys) interaction, and a random intercept for participant.

All analyses were conducted in R Version 4.5.1 (R Core Team 2025) using the packages *lme4* (Version 1.1.37; Bates et al. 2015) and *lmerTest* (Version 3.1.3; Kuznetsova et al. 2017). For the linear mixed‐effects model, significant interactions were followed up with Bonferroni pairwise comparisons using the *emmeans* package (Version 1.11–2.8; Lenth 2025). For the logistic mixed‐effects models, significant interactions were followed up by calculating predicted responses at different levels of the predictor variables using the *ggeffects* package (Version 2.3.1; Lüdecke 2018).

## 3 Results

### 3.1 Judgments of Inequality

The linear mixed‐effects regression model revealed that the main effect of gender on participants’ judgments of inequalities was not significant, *F*(1, 136.58) = 1.66, *p* = 0.205; female participants (*M* = 2.42, *SD* = 1.57) did not judge inequalities significantly more negatively compared to male participants (*M* = 2.67, *SD* = 1.76), thus not supporting hypothesis H1.

**FIGURE 3:**
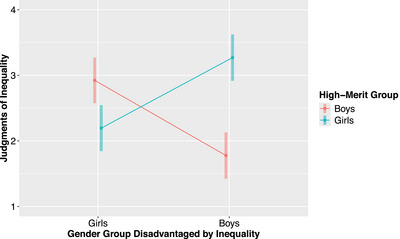
Effect of gender group disadvantage on judgments of inequality by high‐merit group. *N* = 143. The interaction between the gender group disadvantaged by inequality and high‐merit group was significant (*p* < 0.001). The y‐axis represents a subsection of the judgments of inequality scale (1 = really not okay, 4 = a little okay). Error bars represent 95% confidence intervals.

The interaction between the gender group disadvantaged by inequality and high‐merit group was significant, *F*(1, 141.46) = 44.08, *p* < 0.001 (Figure 3). Confirming hypothesis H2, participants overall judged inequalities more negatively when the disadvantaged group was high‐merit compared to low‐merit. Specifically, when girls were disadvantaged, participants judged inequalities that disadvantaged high‐merit girls (*M* = 2.19, *SD* = 1.61) significantly more negatively than inequalities that disadvantaged low‐merit girls (*M* = 2.93, *SD* = 1.61) (*p* = 0.002). When boys were disadvantaged, participants judged inequalities that disadvantaged high‐merit boys (*M* = 1.78, *SD* = 1.27) significantly more negatively than inequalities that disadvantaged low‐merit boys (*M* = 3.27, *SD* = 1.75) (*p* < 0.001).

The interaction between age group and gender group disadvantaged by inequality was not significant, *F*(2, 136.56) = 2.85, *p* = 0.061. However, given our central hypotheses about age group differences for judgments of inequalities that disadvantaged girls (H3 and H4), follow‐up Bonferroni pairwise comparisons were conducted. This revealed that older children's judgments of inequalities that disadvantaged girls (*M* = 2.50, *SD* = 1.49) were not significantly more negative than younger children's judgments (*M* = 3.29, *SD* = 2.00) (*p* = 0.061), but young adults’ judgments of inequalities that disadvantaged girls (*M* = 1.90, *SD* = 0.99) were significantly more negative than younger children's judgments (*p* < 0.001). Thus, hypothesis H3 was partially supported. Young adults’ judgments of inequalities that disadvantaged girls were not significantly more negative compared to older children (*p* = 0.186), thus not supporting hypothesis H4.

Although we found support for hypothesis H2 such that, when both girls and boys were the disadvantaged group, participants judged inequalities more negatively when the disadvantaged group was high‐merit compared to low‐merit, the magnitude of the difference between judgments of inequalities that disadvantaged high‐ and low‐merit boys was twice as large as the difference between judgments of inequalities that disadvantaged high‐ and low‐merit girls (1.49 and 0.74, respectively). To explore how age group may have contributed to this finding, we fit a linear mixed‐effects regression model for judgments of inequality that included a three‐way interaction between age group, gender group disadvantaged by inequality, and high‐merit group, as well as a random intercept for participant. This model revealed that the three‐way interaction was significant, *F*(2, 137.68) = 3.90, *p* = 0.023.

**FIGURE 4:**
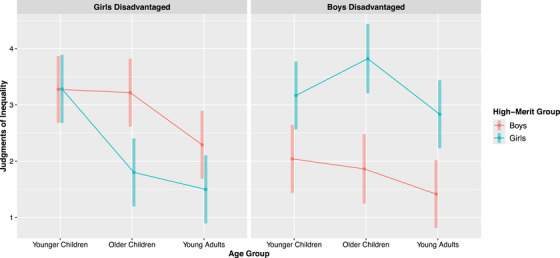
Effect of age group on judgments of inequality by gender group disadvantaged and high‐merit group. *N* = 143. The interaction between age group, gender group disadvantaged by inequality, and high‐merit group was significant (*p* = 0.023). The y‐axis represents a subsection of the judgments of inequality scale (1 = really not okay, 4 = a little okay). Error bars represent 95% confidence intervals.

Follow‐up Bonferroni pairwise comparisons on the three‐way interaction revealed that, when boys were disadvantaged, all age groups judged the inequality more negatively when boys were high‐merit compared to low‐merit (*p*s < 0.05). When girls were disadvantaged, however, only older children judged the inequality more negatively when girls were high‐merit (*M* = 1.79, *SD* = 1.06) compared to low‐merit (*M* = 3.21, *SD* = 1.53) (*p* = 0.004). Younger children's judgments of inequalities that disadvantaged girls did not significantly differ, whether girls were high‐merit (*M* = 3.29, *SD* = 2.12) or low‐merit (*M* = 3.28, *SD* = 1.93) (*p* = 1.00). Young adults’ judgments of inequalities that disadvantaged girls also did not significantly differ, whether girls were high‐merit (*M* = 1.50, *SD* = 0.66) or low‐merit (*M* = 2.29, *SD* = 1.12) (*p* = 0.309) (Figure 4).

Pairwise comparisons also revealed that both older children (*M* = 1.79, *SD* = 1.06) and young adults (*M* = 1.50, *SD* = 0.66) judged inequalities that disadvantaged girls significantly more negatively than did younger children (*M* = 3.29, *SD* = 2.17) (*p*s < 0.05), but only when girls were the high‐merit group. There were no significant age group differences in judgments of inequalities that disadvantaged girls when they were the low‐merit group (*p*s > 0.05).

### 3.2 Reasoning for Judgments of Inequality

#### 3.2.1 Equality

**FIGURE 5:**
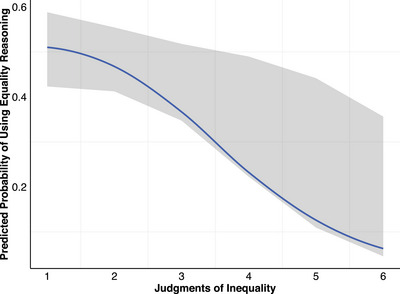
Effect of judgments of inequality on predicted probability of using equality reasoning. *N* = 143. The effect of judgments of inequality on the use of equality reasoning was significant (*p* < 0.001). The x‐axis represents the judgments of inequality scale (1 = really not okay to 6 = really okay). The line represents the predicted probability of using equality reasoning. The shaded region represents the 95% confidence interval.

The logistic mixed‐effects regression model revealed a significant main effect of judgments of inequality on use of equality reasoning, *b* = −0.78, *SE* = 0.15, *p* < 0.001, *OR* = 0.46 (Figure 5). Supporting hypothesis H5a, the more positively participants judged inequalities, the less likely they were to use equality reasoning to justify their judgments.

The interaction between gender group disadvantaged by inequality and high‐merit group was also significant, *b* = 3.33, *SE* = 0.81, *p* < 0.001, *OR* = 27.81. Participants were more likely to use equality reasoning to justify their judgments of inequality when the low‐merit group was disadvantaged compared to the advantaged, thus supporting hypothesis H5b.

Although we did not propose a confirmatory hypothesis about the effect of age group on the use of equality reasoning, it was still included in the model. This revealed that there was a significant main effect of age group on the use of equality reasoning. Both younger children, *b* = 1.67, *SE* = 0.55, *p* = 0.003, *OR* = 5.29, and young adults, *b* = 1.67, *SE* = 0.56, *p* = 0.003, *OR* = 5.31, were significantly more likely to use equality reasoning to justify their judgments of inequality compared to older children.

#### 3.2.2 Merit

**FIGURE 6:**
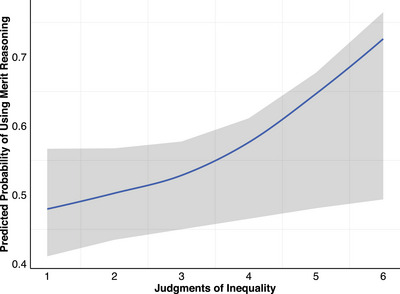
Effect of judgments of inequality on predicted probability of using merit reasoning. *N* = 143. The effect of judgments of inequality on the use of merit reasoning was significant (*p* < 0.001). The x‐axis represents the judgments of inequality scale (1 = really not okay to 6 = really okay). The line represents the predicted probability of using merit reasoning. The shaded region represents the 95% confidence interval.

The logistic mixed‐effects regression model revealed a significant main effect of judgments of inequality on use of merit reasoning, *b* = 0.45, *SE* = 0.13, *p* < 0.001, *OR* = 1.57 (Figure 6). Supporting hypothesis H6a, the more positively participants judged inequalities, the more likely they were to use merit reasoning to justify their judgments.

The interaction between gender group disadvantaged by inequality and high‐merit group was also significant, *b* = −3.12, *SE* = 0.78, *p* < 0.001, *OR* = 0.04. Participants were more likely to use merit reasoning to justify their judgments of inequality when the high‐merit group was disadvantaged compared to the advantaged, thus supporting hypothesis H5b.

Although we did not propose a confirmatory hypothesis about the effect of age group on the use of merit reasoning, it was still included in the model. This revealed that there was a significant main effect of age group on the use of merit reasoning. Both younger children, *b* = −1.87, *SE* = 0.56, *p* < 0.001, *OR* = 0.15, and young adults, *b* = −1.30, *SE* = 0.54, *p* = 0.010, *OR* = 0.25, were significantly less likely to use merit reasoning to justify their judgments of inequality compared to older children.

## 4 Discussion

The present study revealed novel findings regarding the developmental origins of meritocratic beliefs as a social‐cognitive framework that individuals use to evaluate the fairness of inequalities. Specifically, the extent to which younger children, older children, and young adults incorporated concerns for merit into their moral judgments of gender‐based inequalities of science education resources was investigated. Overall, children and young adults judged these inequalities negatively, but they judged inequalities more negatively when groups who displayed high levels of merit (i.e., effort and interest) in science were disadvantaged by inequalities compared to when groups who displayed low levels of merit were disadvantaged by inequalities. Notably, this pattern emerged when both girls and boys were disadvantaged by inequalities.

While these findings align with the wealth of prior research demonstrating children's early emerging preferences for rewarding merit in resource allocation contexts (Baumard et al. 2012; Noh et al. 2019; Schmidt et al. 2016), it is somewhat surprising that participants’ moral judgments were sensitive to information about merit, given the type of resource involved in the inequalities. The science education resources depicted in this study represented a type of necessary resource with implications for children's well‐being, namely their access to learning and cognitive development. Previous research has found that children endorse equal rights to education and negatively evaluate the denial of access to education beginning in late childhood (Helwig and Jasiobedzka 2001; Peterson‐Badali et al. 2004). Yet, despite these views of education, the present finding suggests that children and young adults believed that showing merit, specifically effort and interest, in school made individuals more deserving of educational resources.

This finding extends prior knowledge of how children incorporate concerns for merit into their judgments of necessary resource allocations. Rizzo and colleagues (Rizzo et al. 2016) demonstrated that children judged merit‐based allocations of necessary (i.e., health‐related) resources more negatively than merit‐based allocations of luxury (i.e., play‐related) resources, therefore suggesting that children recognized the implications that unequal allocations of necessary resources can have on others’ well‐being. The present study suggests that children's coordination of concerns for merit and others’ well‐being is more nuanced. Children, as well as young adults, generally viewed all science education resource inequalities negatively, demonstrating an awareness of the implications that being deprived of these important resources can have on individuals. However, they still incorporated concerns for merit into their views about what was fair in this context in a significant way, as reflected by their more negative judgments of inequalities that disadvantaged high‐merit groups compared to low‐merit groups.

Children's relative acceptance of inequalities that disadvantaged low‐merit groups compared to high‐merit groups provides novel insights into the developmental origins of adults’ well‐documented justification of social inequalities with meritocratic beliefs (Jost et al. 2015; Ledgerwood et al. 2011; Mijs 2021). Specifically, participants in this study were presented with information about the merit of different groups, and all members of the groups were described as having the same level of merit. This homogenization of the traits of all members from certain groups mirrors the structure of stereotypes, as they reduce the variability between individuals and instead link their traits solely to their group membership. Participants then factored this information about groups’ merit into their moral judgments of inequalities and viewed it as more acceptable to give fewer resources to groups who were low‐merit compared to high‐merit.

The application of information about groups’ merit to participants’ inequality judgments closely parallels the ways in which meritocratic beliefs can interact with stereotypes to lead individuals to justify social inequalities (Madeira et al. 2019, McCoy and Major 2007). In this study, children and young adults believed the information they were given about different groups’ merit levels to be true, as they had no reason to believe otherwise. This experimental paradigm offered a single dose of what children often experience as they move through the world and encounter messages from peers, parents, teachers, and media that promote stereotypic beliefs about different social groups. If individuals internalize these messages and personally endorse stereotypes about specific social groups’ traits and abilities, they may draw upon them to make sense of instances in which those groups are disadvantaged by inequalities, as was found in the current study.

This finding enriches the growing body of research on children's tendencies to attribute inequalities to internal causes (i.e., differences in effort, abilities, etc.) when information about structural causes is absent. Prior work has suggested that children may default to internal rather than structural explanations for inequalities due to their ignorance about the sociohistorical context of inequalities, the relatively lower cognitive demands of internal explanations, and their essentialist views of social groups (Hussak and Cimpian 2015; Rhodes and Mandalaywala 2017; Zhang et al. 2024). However, this study offers an additional explanation, which is that children's early emerging sensitivity to merit in their conceptions of fairness contributes to their affinity for internal inferences about inequalities. Thus, the present study represents a novel integration of developmental science research on children's preferences for merit in resource allocation contexts (Baumard et al. 2012; Noh et al. 2019; Schmidt et al. 2016) with social psychological research and sociological theory on the justification of social inequalities with meritocratic beliefs (Bonilla‐Silva 2022; Jost et al. 2015; Mijs 2021) to further understand the social‐cognitive processes that shape individual's developing perspectives on social inequalities.

Given that strong cultural stereotypes about girls’ lack of interest and ability in STEM fields emerge early in development and persist into adulthood (Cheryan et al. 2017; Master et al. 2021; McGuire et al. 2022), it is interesting that participants overall incorporated the information about merit into their inequality judgments when both girls and boys were disadvantaged by inequalities. However, exploratory analyses revealed that the extent to which participants incorporated information about girls’ and boys’ merit into their inequality judgments varied by age group. Specifically, when boys were disadvantaged by inequalities, all age groups viewed it as less acceptable to disadvantage boys when they were high‐merit compared to low‐merit. Yet, when girls were disadvantaged by inequalities, only older children viewed it as less acceptable to disadvantage girls when they were high‐merit compared to low‐merit. Younger children's and young adults’ judgments of inequalities that disadvantaged girls did not differ significantly based on girls’ merit level.

Notably, younger children's judgments of inequalities that disadvantaged both high‐ and low‐merit girls were relatively neutral (3.29 and 3.28, respectively, on a 6‐point scale). This suggests that younger children did not view giving girls fewer science resources than boys as much of a moral issue, regardless of how much merit they displayed. One possible explanation for these relatively neutral judgments is that younger children may have endorsed stereotypes about girls’ STEM‐related interests and abilities, which have been found to develop around the ages of 5 to 6 years old (Bian et al. 2017; Master et al. 2021; McGuire et al. 2022). If younger children believed girls to generally be less interested in and good at science than boys, they may have believed that giving them fewer science resources was only slightly wrong. This finding is somewhat concerning, as it suggests that explicit information about girls’ effort and interest in science may not be enough to counteract young children's stereotypic beliefs and influence them to view inequalities that disadvantage girls in STEM contexts as serious moral transgressions.

Conversely, young adults’ judgments of inequalities that disadvantaged both high‐ and low‐merit girls were very negative (1.50 and 2.29, respectively, on a 6‐point scale). These negative judgments suggest that young adults viewed giving fewer science resources to girls as a serious moral transgression, regardless of their level of merit. Young adults may have prioritized their knowledge of girls as a societally disadvantaged group in STEM to judge all inequalities that disadvantaged girls negatively. That is, even if the girls in the vignettes did not work hard and show interest in science, young adults may have viewed it as unfair to give them fewer science resources because girls have historically been disadvantaged in STEM contexts on a broader societal level. This interpretation fits with previous research guided by the SRD model showing that, with age, individuals can apply knowledge about past intergroup relations and group status to evaluate unfair treatment against historically disadvantaged groups as particularly wrong (Elenbaas et al. 2020; Herry et al. 2023; Killen et al. 2024).

Despite the alignment of these age‐related findings with prior theory and research, they should be interpreted with caution due to the relatively small cell sizes of the three‐way interaction (i.e., approximately 25 participants from each age group in each between‐subjects condition). Furthermore, although there is theoretical support for the interpretations of why younger children and young adults appeared not to prioritize information about merit in their judgments of inequalities that disadvantaged girls, future research could strengthen these interpretations by directly assessing the statistical relationships between participants’ endorsement of gender STEM stereotypes, perceptions of gender and status in STEM, and inequality judgments.

Children's and young adults’ reasoning for their inequality judgments also yielded novel findings. Specifically, age‐related differences in the usage of distinct moral reasoning categories emerged such that older children were more likely to use merit reasoning, and less likely to use equality reasoning, to justify their judgments of inequalities compared to both younger children and young adults. These reasoning findings reflect the sensitivity to merit that was found in older children's judgments of inequalities that disadvantaged both boys and girls and are consistent with previous research highlighting this developmental period as a time where effort and positive intentions are highly salient in resource inequality contexts (Noh et al. 2019; Schmidt et al. 2016).

Additionally, this study yielded novel evidence of the relationship between moral judgments and reasoning that has been found in previous SRD research. Specifically, in line with prior research (Rizzo et al. 2016; Rizzo and Killen 2020), the present study found that the more positively participants judged inequalities, the less likely they were to use equality reasoning to justify their judgments. Conversely, the more positively participants judged the inequalities, the more likely they were to use merit reasoning to justify their judgments. These findings are particularly relevant to the SRD model's utility to understand developing perspectives on fairness and equality in STEM contexts, where both merit and gender group membership are important factors that contribute to inequalities (Blair‐Loy and Cech 2022; Cheryan et al. 2025; Mulvey et al. 2023).

### 4.1 Limitations and Future Directions

The present study provided novel insights into the developmental origins of meritocratic beliefs and how they can be applied to judgments of necessary resource inequalities. However, there are limitations to the current study that provide a basis for future research. The focus of the current study was on individuals’ judgments and reasoning about gender‐based science resource inequalities, and thus, one area for future research is to investigate individuals’ responses to these inequalities. Examining responses to inequalities can provide more nuanced information about individuals’ conceptions of equality and equity (Rizzo et al. 2020; Rutland and Killen 2017), which is especially important to examine in STEM because achieving gender equity necessitates acknowledging past inequalities. Additionally, future research exploring individuals’ responses to gender‐based science resource inequalities may help reconcile the finding that young adults judged inequalities that disadvantaged girls in the vignettes quite negatively, yet gender STEM inequalities remain pervasive in adulthood (Cheryan et al. 2025).

This study specifically investigated judgments and reasoning about science resource inequalities between groups of boys and girls attending different schools. However, these gender‐segregated settings do not represent many children's experiences of mixed‐gender classes in school or adults’ workplace experiences. Thus, an important avenue for future research is to explore how individuals coordinate concerns for merit and group membership when assessing gender‐based inequalities that exist *within* a mixed‐gender group. One way to explore this question is to assess judgments of inequalities of opportunities within a group (e.g., leadership positions). Such scenarios would more accurately represent how gender STEM inequalities often unfold in everyday life and may also tap into group norms and group dynamics to a greater extent than the current study did.

Finally, the present study was strengthened by the inclusion of participants from several different developmental periods, but adolescents were not represented in the sample. Adolescence is a critical time for the development of factors that promote interest in STEM careers, such as STEM identity and belonging (Kim et al. 2018; Master et al. 2016), and it is also a period during which individuals’ capacities to apply their knowledge about group status to their moral judgments and decision‐making increases (Killen et al. 2024; Yüksel et al. 2021). Given that the exploratory age‐related findings showed that older children, but not young adults, incorporated information about merit into their judgments of inequalities that disadvantaged girls, it is important for future research to include adolescents to more precisely identify when the transition from merit‐driven judgments in late‐childhood to equity‐driven judgments in young adulthood occurs.

## 5 Conclusion

This study demonstrates novel evidence of the development of meritocratic beliefs and their application to judgments of group‐based inequalities. Children and young adults generally viewed all inequalities of science education resources as wrong, but they viewed inequalities that disadvantaged groups who showed high merit in science as especially wrong. While this pattern emerged across participants overall when both girls and boys were disadvantaged by inequalities, exploratory findings suggest that the extent to which individuals incorporate merit into their judgments of inequalities that disadvantage girls, a historically disadvantaged group in STEM contexts, varies with age. Older children showed a unique sensitivity to concerns for merit in both their judgments and reasoning about inequalities. Together, these findings advance theory and research on developmental conceptions of fairness and hold implications for how social inequalities, such as gender inequalities in STEM, are addressed throughout childhood and young adulthood.

## Conflicts of Interest

The authors declare no conflicts of interest.

## Data Availability

This study was not pre‐registered; data and code will be available upon request from the corresponding author.
